# Allostatic Load and Cognitive Function Among Black and White Participants in the REGARDS Cohort Study

**DOI:** 10.1002/alz70860_102271

**Published:** 2025-12-23

**Authors:** Ambar Kulshreshtha, Alvaro Alonso, Ganesh M. Babulal, Suzanne E Judd

**Affiliations:** ^1^ Emory University School of Medicine, Atlanta, GA, USA; ^2^ Emory University Rollins School of Public Health, Atlanta, GA, USA; ^3^ Washington University School of Medicine, Saint Louis, MO, USA; ^4^ School of Public Health, University of Alabama at Birmingham, Birmingham, AL, USA

## Abstract

**Background:**

The cumulative impact of stress on the body has long‐term consequences on cognition. Allostatic load (AL) is an important composite metric for quantifying biological risk and health implications arising from the physiological dysregulation caused by chronic stress. We examined the longitudinal association between AL and cognitive impairment (CI).

**Methods:**

The REasons for Geographic And Racial Differences in Stroke (REGARDS) is a population‐based cohort of 30,239 Black and White participants over 45 years old with ongoing follow‐up. Participants were recruited from 2003‐2007, and data was collected through telephone, self‐administered questionnaires, and an in‐home examination. Cognitive function was assessed with the six‐item screener (SIS), and participants with a score ≤ 4 were considered to have CI. To assess AL, a numerical score of 0, 0.5, or 1 was allocated to the respective low, moderate, and high‐risk classifications for each biomarker (composite of lipid, blood pressure, blood glucose, C‐reactive protein, body mass index, albumin levels, glomerular filtration rate). We explored effect modification by race, sex, and age. Logistic regression provided estimates for odds ratios (ORs) for prevalent CI at baseline and incident CI at the most recently observed follow‐up with SIS by tertiles of AL.

**Results:**

There were 1783 participants included in the final sample. The average age was 67 years, 41% were Black. The highest AL tertile had a higher proportion of male participants, people with lower incomes and lower education, and those who self‐identified as Black. The mean AL was 3.29 (SD =0.79, range 1.5‐6.5). AL was not associated with prevalent CI (Tertile 3 vs 1: OR 1.12, 95% CI 0.78, 1,60). In the longitudinal analyses, higher AL was associated with incident CI after adjusting for multiple potential confounders (Tertile 3 vs 1: OR=1.26, 95% CI 1.07,1.48). There was significant interaction with age, with younger participants (<65 years) having a stronger association with AL and CI than older participants (OR 3.9, 95% CI 1.59‐9.56) vs (OR 0.88, 95% CI 0.63, 1.24).

**Conclusion:**

AL was associated with incident CI in adults <65 years old, suggesting that younger individuals may be more vulnerable to the effects of elevated AL on cognitive function.

